# Primary health care for people experiencing homelessness: the effectiveness of specialist and mainstream health service provision

**DOI:** 10.3399/bjgp24X740217

**Published:** 2024-11-29

**Authors:** Maureen Crane, Louise Joly, Blánaid JM Daly, Heather Gage, Jill Manthorpe, Gaia Cetrano, Chris Ford, Peter Williams

**Affiliations:** NIHR Policy Research Unit in Health and Social Care Workforce, King’s College London, London, UK.; NIHR Policy Research Unit in Health and Social Care Workforce, King’s College London, London, UK.; Trinity College Dublin, Dublin, Ireland.; Surrey Health Economics Centre, Department of Clinical and Experimental Medicine, University of Surrey, Guildford, UK.; NIHR Policy Research Unit in Health and Social Care Workforce, King’s College London, London, UK.; Middlesex University, London, UK.; London, UK.; Department of Mathematics, University of Surrey, Guildford, UK.

## Introduction

Homelessness is a growing problem in many high-income countries, and physical health, mental health, and substance misuse problems are common among people affected.^1^^,^^2^ Many have multiple long-term conditions and their health needs are often greater than those of the general population. Since the 1970s, specialist primary healthcare services for people experiencing homelessness developed in the UK and elsewhere, generating debates about the benefits and drawbacks of specialist versus mainstream approaches.^3^^,^^4^ There have been few UK studies of the effectiveness of specialist provision, with the Department of Health in 2010 identifying a need for such evidence.^5^ The HEARTH study (2015–2023) aimed to address this knowledge gap by comparing the effectiveness of four primary healthcare models in England used by people experiencing homelessness. The models, identified by an initial mapping survey,^6^^,^^7^ were: 1) health centres primarily for people experiencing homelessness (Dedicated Centres); 2) mobile health teams that hold clinics in homelessness sector hostels or day centres (Mobile Teams); 3) mainstream general practices providing additional services for patients experiencing homelessness (Specialist GPs); and 4) mainstream general practices providing general medical services but no additional services for those experiencing homelessness (Usual Care GPs).

This article critically reviews these four models, the features of which are summarised in Table 1. HEARTH recruited 96 participants (single people experiencing homelessness) for each of the three specialist models (from two sites per model) and 75 participants for the Usual Care GP model (from four general practices). Participants’ characteristics are summarised in Table 2. Measures of effectiveness for the models included: 1) health screening for six indicators with an intervention if required; 2) continuity of care for four long-term or complex health conditions; 3) participants’ use of health services over the 12-month study period; and 4) participants’ satisfaction with the care they received. Outcomes are summarised in Table 3. A full description of the study design, methods, analyses, and results is available in the study report.^9^ The implications for NHS commissioners, primary care managers, and staff are outlined in briefing papers.^10^^,^^11^

**Table 1.** Key characteristics of the Health Service Models

**Characteristics**	**Dedicated Centres**	**Mobile Teams**	**Specialist GPs**	**Usual Care GPs**
Number of sites	2	2	2	4
Service delivered from fixed NHS premises	Yes	No	Yes	Yes
Primarily for people who are homeless	Yes	Yes	No	No
Number of patients registered	<600 & <1400	<200 & <1400	>5000 at each site	>10 000 at each site
Patients who are currently or recently homeless, %	100	100	4–7	<5
Has GPs/provides GP registration	Yes	No	Yes	Yes
*Specific staff at the sites*				
Nurses primarily for patients who are homeless	Yes	Yes	1 site	No
Mental health nurse/psychiatrist at least weekly	Yes	1 site	1 site	1 site
Substance misuse worker at least weekly	Yes	No	1 site	2 sites
Social workers/case workers	Yes	No	1 site	No
*Drop-in clinics/outreach*				
Drop-in clinics at the study site primarily for people who are homeless	Yes	Yes	1 site	No
Clinics in hostels/day centres	1 site	Yes	Yes	No
Outreach on streets	Yes	Yes	1 site	No
Hospital liaison/discharge work for patients who are homeless	Yes	Yes	1 site	No

**Table 2.** Key characteristics of study participants by Health Service Model

**Characteristics at baseline**	**All, *N* = 363^a^**	**Dedicated Centre, *N* = 96**	**Mobile Team, *N* = 96**	**Specialist GP, *N* = 96**	**Usual Care GP, *N* = 75**	**Comparison test: *P*-value**
Male, *n* (%)	291 (80.2%)	77 (80.2%)	75 (78.1%)	71 (74.0%)	68 (90.7%)	χ^2^: 0.051
Age in years, mean (SD)	41.6 (10.5)	39.8 (9.8)	43.0 (11.3)	41.1 (9.9)	42.6 (11.0)	ANOVA: 0.147
Born in the UK (*N* = 362), *n* (%)	289 (79.8)	83 (86.5)	53 (55.2)	92 (96.8)	61 (81.3)	χ^2^: <0.001
Years homeless (*N* = 359), median (IQR)	5.5 (2.5–12.0)	5.5 (2.2–11.0)	5.7 (2.0–11.4)	5.1 (2.4–12.5)	6.5 (3.0–12.5)	Kruskal–Wallis: 0.478
Physical health problems^b^ (*N* = 362), *n* (%)	343 (94.8)	90 (94.7)	91 (94.8)	91 (94.8)	71 (94.7)	χ^2^: 0.987
Mental health problems^b^ (*N* = 361), *n* (%)	329 (91.1)	88 (91.7)	79 (84.0)	88 (91.7)	74 (98.7)	χ^2^: 0.011
**In 4 months prior to baseline interview**						
Harmful alcohol drinking,^b,c^ (*N* = 345), *n* (%)	110 (31.9)	26 (29.5)	28 (30.4)	32 (34.4)	24 (33.3)	χ^2^: 0.884
Misused drugs,^b,d^ (*N* = 361), *n* (%)	217 (60.1)	59 (62.1)	44 (45.8)	67 (69.8)	47 (63.5)	χ^2^: 0.006
Used Class A drugs,^b, e^ (*N* = 361), *n* (%)	146 (40.4)	46 (48.4)	23 (24.0)	54 (56.3)	23 (31.1)	χ^2^: <0.001
**During 12-month study period**						
Percentage of time in building designed for habitation^f^ (*N* = 360), mean (SD)	79.78 (30.62)	78.61 (29.20)	63.68 (37.20)	83.01 (27.36)	97.38 (10.30)	ANOVA: <0.001
Percentage of time in accommodation with staff^g^ (*N* = 360), mean (SD)	48.21 (39.07)	35.94 (35.85)	44.86 (36.69)	50.71 (36.46)	64.93 (43.32)	ANOVA: <0.001
Number of changes of accommodation/sleeping settings,^h^ mean (SD)	2.29 (2.32)	2.77 (2.61)	2.55 (2.06)	2.47 (2.48)	1.13 (1.60)	ANOVA: <0.001

a *As shown in column 1, N is less than 363 for some characteristics because of missing data. The numbers/percentages in each column relate only to participants in each model who responded to the question and reported the characteristic.*

b *Self-reports.*

c *35+ units weekly for women, and 50+ units weekly for men.^8^*

d *Illegal drugs or misuse of prescription or over-the-counter drugs.*

e *Under the Misuse of Drugs Act 1971, Class A drugs include heroin, morphine, cocaine, LSD, and ecstasy.*

f *Hostel, supported housing, bed-and-breakfast hotel, or rented accommodation. Not sleeping on the streets, or in a tent, vehicle, squat, or church hall.*

g *24-hour or part-time staff.*

h *For example, a move from the streets to a hostel, or from a hostel to a bed-and-breakfast hotel or to the streets. ANOVA = analysis of variance. IQR = interquartile range.*

*SD = standard deviation.*

**Table 3.** Study outcomes by Health Service Model

**Outcomes**	**All, *N* = 363**	**Dedicated Centre, *N* = 96**	**Mobile Team, *N* = 96**	**Specialist GP, *N* = 96**	**Usual Care GP, *N* = 75**	**Comparison test: *P*-value**
**Health screening^a,b^**						
Number of indicators screened (*N* = 349), median (minimum, maximum)	3.00 (0, 6)	3.50 (1, 6)	3.00 (0, 6)	3.50 (0, 5)	4.00 (1, 6)	Kruskal–Wallis: 0.002
Number of indicators screened (*N* = 349), mean (SD)	3.30 (1.24)	3.54 (1.03)	2.90 (1.41)	3.34 (1.24)	3.51 (1.11)	Not applicable; defer to the row above
**Continuity of care for long-term health conditions over 12-month study period^a, c,d^**						
Chronic respiratory problems (*N* = 65), *n* (%)	46 (70.8)	14 (87.5)	7 (46.7)^e^	19 (76.0)	6 (66.7)	χ^2^: 0.079
Depression (*N* = 156), *n* (%)	90 (57.7)	35 (70.0)	12 (34.3)^e^	31 (64.6)	12 (52.2)	χ^2^: 0.007
Alcohol problems (*N* = 153), *n* (%)	83 (54.2)	32 (72.7)	11 (29.7)^e^	27 (61.4)	13 (46.4)	χ^2^: 0.001
Drug problems (*N* = 177), *n* (%)	96 (54.2)	45 (84.9)	9 (25.7)^e^	39 (56.5)	3 (15.0)	χ^2^: <0.001
**Contacts with primary care staff over 12-month study period^a,f^**						
GP contacts (*N* = 363), median (IQR)	7.0 (4.0–14.0)	14.0 (7.0–21.7)	6.0 (2.0–11.0)	7.5 (4.0–14.0)	4.00 (2.0–7.0)	Kruskal–Wallis: <.001
Primary care nurses/HCA contacts (*N* = 363), median (IQR)	3.0 (1.0–9.0)	3.0 (1.0–8.7)	13.0 (7.0–21.0)	1.0 (0.0–4.0)	2.0 (1.0–4.0)	Kruskal–Wallis: <.001
**Participants’ perspectives at baseline of study site**						
Overall experience of study site (*N* = 355), median (IQR) rating^g^	1.0 (1.0–2.0)	1.0 (1.0–2.0)	1.0 (1.0–1.0)	1.0 (1.0–2.0)	2.0 (1.0–2.0)	Kruskal–Wallis: <.001
Confidence/trust in site GP when last seen (*N* = 207), n (%)^h^						
Yes, definitely	157 (75.8)	61 (82.4)	N/A	67 (81.7)	29 (56.9)	Kruskal–Wallis: 0.001
Yes, to some extent	25 (12.1)	6 (8.1)		9 (11.0)	10 (19.6)	
Not at all	25 (12.1)	7 (9.5)		6 (7.3)	12 (23.5)	

a *Data from medical records; the records were not obtained for 14 Usual Care GP participants.*

b *Six indicators examined: record of 1) BMI, alcohol use, mental health assessment, TB screening, in last 12 months; 2) smoking status in last 24 months; and 3) hepatitis A vaccine or immunity in last 10 years.*

c *Different criteria for each condition drawing on QOF indicators and expert advisers to the study, for example, for alcohol problems, 3-monthly reviews by study site staff, or feedback from alcohol service.*

d *Column 1 refers to number of participants who reported the health condition at their baseline interview and it was documented in their medical records during the first 4 months of the study. Subsequent columns relate to the number/percentage in each model with the condition who received continuity of care.*

e *When interventions by GPs were included in Mobile Teams’ continuity of care ratings, scores reached: 60.0% for chronic respiratory problems, 52.2% for depression, 46.4% for alcohol problems, and 56.5% for drug problems.*

f *At study site, clinics in hostels and day centres, other sites, and telephone consultations.*

g *Rating with lower scores more positive: 1 = very good; 2 = fairly good; 3 = neither good nor poor; 4 = fairly poor; 5 = very poor.*

h *Only includes participants of each model who answered the question and saw a GP (not a nurse) on their last visit. BMI = body mass index HCA = healthcare assistant. IQR = interquartile range. N/A = not applicable. QOF = Quality and Outcomes Framework.*

## Model 1: Dedicated Centres

The mapping identified 28 Dedicated Centres across England, most of which were in large towns or cities. Although the study participants from the two sites represented in HEARTH tended to be less settled and had more complex needs than those of Mobile Teams and Usual Care GPs, their outcomes were consistently among the best. There was no statistically significant difference between this model and Specialist GPs and Usual Care GPs in health screening (Mobile Teams scored significantly lower). However, Dedicated Centres scored higher than other models in continuity of care for patients with depression and substance misuse problems. Their participants also had substantially more GP contacts during the study period and, apart from Mobile Team participants, more nurse contacts.

Several contextual and service delivery factors are likely to have contributed to the model’s relative success. Both sites had been in operation for years, most staff had considerable experience of working with this patient group, and their focus was primarily on people who were homeless. The number of registered patients was notably smaller than that of the other GP-led models, allowing the delivery of flexible, holistic, and well-integrated care, such as daily drop-in clinics, longer than customary GP consultations, and street outreach. Multidisciplinary working was a key factor at both sites, with social workers/case workers employed as core staff alongside GPs and practice nurses, on-site clinics run by mental health and substance misuse workers, and close working with homelessness sector services and local hospitals.

## Model 2: Mobile Teams

Twelve mobile homeless health teams were identified during the mapping, two of which represented this model in HEARTH. The two HEARTH teams mainly consisted of specialist nurse practitioners and one employed a part-time mental health nurse. Neither team included a GP and patients were therefore encouraged to register with a local general practice. Compared with other models, Mobile Team participants were more likely to have been born outside the UK and less likely to report drug misuse and mental health problems. This model had less favourable scores for health screening and continuity of care for long-term health conditions. However, when interventions by GPs were included in continuity of care ratings, scores reached levels comparable with or above those of Usual Care GPs but lower than those of Dedicated Centres and Specialist GPs. The mean number of nurse consultations during the study period was considerably higher for Mobile Team participants, although they had fewer GP contacts than Dedicated Centre and Specialist GP participants.

Service delivery factors are likely to have affected outcomes. This model operated as a ‘Mobile Team plus separate GP model’ with patients receiving health care from *two* separate services at *different* sites. Participants in each Mobile Team were not registered at the same GP practice, and therefore the Mobile Team nurses needed to coordinate care with several general practices. They worked closely with some GPs, held some joint clinics, and shared medical records. However, lower health screening and continuity of care scores suggest poor coordination between the two services. Furthermore, the Mobile Team nurses were without ‘fixed’ NHS premises where patients could be seen. They relied on homelessness services to host clinics, were restricted to specific days and times, and facilities ranged from well-equipped medical rooms to screened areas in communal spaces. Although the Mobile Teams were well integrated with homelessness services, there was little joint working with substance misuse teams.

## Model 3: Specialist GPs

The mapping identified 61 GP practices providing enhanced services for people who were homeless, ranging from drop-in clinics at the practice, to clinics at homelessness services and street outreach. The number of patients registered at the two HEARTH sites was considerably higher than that of Dedicated Centres because they were primarily mainstream general practices. There were marked differences between the two sites in this model, with one (SP1) providing more intensive services to people experiencing homelessness, comparable in many ways to Dedicated Centres. It had dedicated nurses, case managers, drop-in clinics, on-site clinics run by mental health and substance misuse workers, and frequent outreach on the streets and in homelessness services. The other site (SP2) had no staff working exclusively for patients who were homeless, no on-site clinics run by substance misuse teams, and fewer outreach activities. Specialist GP study participants’ characteristics were similar to those of Dedicated Centre participants except they were more stably housed during the study.

Overall, the Specialist GP model scored favourably for health screening (mean 3.34) and continuity of care (for example, 56.5% for drug problems), but largely because SP1 achieved a very high rate for health screening (mean 4.16 versus 2.42 for SP2) and higher continuity of care rates (70.3% for SP1 participants with drug problems versus 40.6% at SP2). SP1’s more integrated work is likely to have aided its positive outcomes. In contrast, SP2 staff relied on external substance misuse services to provide treatments and received little feedback about patients’ progress. They explained that insufficient resources prevented them from conducting more outreach and collaborating more with other agencies.

## Model 4: Usual Care GPs

Almost one-half of 753 homelessness sector hostels and day centres in England surveyed during the mapping were *not* served by a specialist primary healthcare service.^6^ Instead, their clients received health care from mainstream GPs. HEARTH participants for the Usual Care GP model were drawn from four practices, which were very large, had no special services for patients experiencing homelessness, little input from mental health and substance misuse teams, and their staff had few links to homelessness sector services. The model scored favourably for health screening but performed less well for continuity of care, especially for drug problems. Participant satisfaction was also poorer, with several participants reporting difficulties booking GP appointments and long waits until their appointment date, and they had lower GP consultation rates than the specialist models. One possible reason for the model’s relatively high health screening score, mainly for smoking, alcohol use, and body mass index recording, is that these indicators are routinely screened by many mainstream general practices in accordance with the English pay-for-performance system (Quality and Outcomes Framework or QOF) and National Institute for Health and Care Excellence (NICE) guidelines.

Compared with other models, Usual Care GP participants tended to be more settled and had less complex needs: most were staying in hostels throughout the study, and were less likely to misuse Class A drugs. Given the difficulties experienced by this model in providing care to a relatively settled population of hostel residents, it is likely that such practices would struggle to deliver a service to greater numbers of people with complex needs and unsettled lifestyles. General practice staff cited insufficient resources as preventing more proactive work with this patient group. Participants’ poorer satisfaction scores and low rate of GP use also indicate their difficulties in accessing care from mainstream general practices.

## Conclusion

Since 2010, homelessness has increased substantially across England, and cuts to housing, health, and support budgets limit the help that can be provided.^12^ New NHS structures (such as local Primary Care Networks of general practices and sub-regional Integrated Care Systems) are central to the future of primary care. Their stated aims include improving patient outcomes and access to health care, and reducing health inequalities.^13^ HEARTH demonstrated the benefits to people experiencing homelessness of targeted and enhanced health services offering flexibility and multidisciplinary working. For example, there was a significant association between provision of on-site substance misuse clinics at the study sites and improved continuity of care for alcohol and drug problems. NICE also stressed the importance of people experiencing homelessness of integrated health and social care, including low-threshold health services.^14^ Many of HEARTH’s findings corroborate US data which demonstrate that tailored and integrated services and outreach bring positive health outcomes for people experiencing homelessness (as noted in the study’s literature review).^7^ More recent literature reviews describe similar findings.^15^^,^^16^

HEARTH found that the Dedicated Centre model performed best for many outcomes. However, because of its uniqueness and specialism, it may only be practically and financially viable in locations with a considerable number of people experiencing homelessness. One Specialist GP site (SP1) offered similar enhanced services, had comparable outcomes, and effectively operated in an urban area with a large homeless population. This suggests that this model might be a more widely viable alternative to the Dedicated Centre model, providing that sufficient resources are available to enable delivery of flexible, targeted, and integrated services to people experiencing homelessness.

Findings from HEARTH raise questions about the Mobile Team model. While potentially reaching people who may be hesitant about accessing health care, mobile teams might be more effective operating as an outreach part of a general practice rather than as a ‘stand-alone’ service. Their patients would be registered with a *single* primary care provider with a fixed base. It would in effect become a Specialist GP model offering improved coordination between GPs and other primary care professionals. The Usual Care GP model is the main type of primary care provision for many people experiencing homelessness, but its relatively poor performance in HEARTH raises concerns about the capacity of mainstream general practices to support patients experiencing homelessness, and the threshold at which additional services are required. The introduction of a ‘homelessness lead’ within such practices might enable more proactive and coordinated work with this patient group, or a more targeted approach may be required such as a Specialist GP model.

Regardless of the model, it is crucial that effective monitoring and evaluation are included in the commissioning of primary healthcare services for people experiencing homelessness. Both the Royal College of General Practitioners and NICE have noted the importance of commissioners being aware of the effectiveness of services and those that are not meeting their objectives.^14^^,^^17^ In HEARTH, use of indicators to assess performance was inconsistent across sites. One solution may be to introduce key performance indicators that encompass health screening, access to treatment, and the management of long-term health conditions to QOF standards, as described by the Faculty for Homeless and Inclusion Health.^18^

## References

[1] Aldridge RW, Menezes D, Lewer D (2019). Causes of death among homeless people: a population-based cross-sectional study of linked hospitalisation and mortality data in England. Wellcome Open Res.

[2] Wright N, Smeeth L, Heath I (2003). Moving beyond single and dual diagnosis in general practice: many patients have multiple morbidities, and their needs have to be addressed. BMJ.

[3] Wright N (2002). Homelessness: a primary care response.

[4] Connelly J, Crown J (1994). Homelessness and ill health.

[5] Department of Health, Office of the Chief Analyst (2010). Healthcare for single homeless people.

[6] Crane M, Cetrano G, Joly L (2018). Mapping of specialist primary health care services in England for people who are homeless.

[7] Crane M, Cetrano G, Joly L (2018). Inventory of specialist primary health care services in England for people who are homeless.

[8] National Institute for Health and Care Excellence (2010). Alcohol-use disorders: prevention PH24.

[9] Crane M, Joly L, Daly BJM (2023). Integration, effectiveness and costs of different models of primary health care provision for people who are homeless: an evaluation study. Health Soc Care Deliv Res.

[10] Crane M, Joly L, Daly BJM (2023). Integration, effectiveness and costs of different models of primary health care provision for people who are homeless: an evaluation study. Findings and implications for NHS primary care and integrated care commissioners.

[11] Crane M, Joly L, Daly BJM (2023). Integration, effectiveness and costs of different models of primary health care provision for people who are homeless: an evaluation study. Findings and implications for NHS primary care managers and practice staff.

[12] National Audit Office (2017). Homelessness.

[13] Waller E, Kanani N (2021). Primary Care Networks — plans for 2021/22 and 2022/23.

[14] National Institute for Health and Care Excellence (2022). Integrated health and social care for people experiencing homelessness NG214.

[15] Jego M, Abcaya J, Ștefan DE (2018). Improving health care management in primary care for homeless people: a literature review. Int J Environ Res Public Health.

[16] Kopanitsa V, McWilliams S, Leung R (2023). A systematic scoping review of primary health care service outreach for homeless populations. Fam Pract.

[17] Gill P, MacLeod U, Lester H, Hegenbarth A (2013). Improving access to health care for Gypsies and Travellers, homeless people and sex workers.

[18] Hewett N (2018). Homeless and Inclusion Health standards for commissioners and service providers.

